# Gut microbiome modulation during treatment of mucositis with the dairy bacterium *Lactococcus lactis* and recombinant strain secreting human antimicrobial PAP

**DOI:** 10.1038/s41598-018-33469-w

**Published:** 2018-10-10

**Authors:** Rodrigo Carvalho, Aline Vaz, Felipe Luiz Pereira, Fernanda Dorella, Eric Aguiar, Jean-Marc Chatel, Luis Bermudez, Philippe Langella, Gabriel Fernandes, Henrique Figueiredo, Aristóteles Goes-Neto, Vasco Azevedo

**Affiliations:** 1 Federal University of Minas Gerais (UFMG-ICB), Belo Horizonte, MG Brazil; 2 Federal University of Bahia (UFBA), Salvador, BA Brazil; 3 Fiocruz - Centro de Pesquisa Renê Rachou, Belo Horizonte, MG Brazil; 4 Micalis Institute, INRA, AgroParisTech, Université Paris-Saclay, Jouy-en-Josas, France

## Abstract

Mucositis is an inflammatory condition of the gut, caused by an adverse effect of chemotherapy drugs, such as 5-fluorouracil (5-FU). In an attempt to develop alternative treatments for the disease, several research groups have proposed the use of probiotics, in particular, Lactic Acid Bacteria (LAB). In this context, the use of recombinant LAB, for delivering anti-inflammatory compounds has also been explored. In previous work, we demonstrated that either *Lactococcus lactis* NZ9000 or a recombinant strain expressing an antimicrobial peptide involved in human gut homeostasis, the Pancreatitis-associated Protein (PAP), could ameliorate 5-FU-induced mucositis in mice. However, the impact of these strains on the gut microbiota still needs to be elucidated. Therefore, in the present study, we aimed to characterize the effects of both *Lactococci* strains in the gut microbiome of mice through a 16 S rRNA gene sequencing metagenomic approach. Our data show 5-FU caused a significant decrease in protective bacteria and increase of several bacteria associated with pro-inflammatory traits. The *Lactococci* strains were shown to reduce several potential opportunistic microbes, while PAP delivery was able to suppress the growth of *Enterobacteriaceae* during inflammation. We conclude the strain secreting antimicrobial PAP was more effective in the control of 5-FU-dysbiosis.

## Introduction

Oncology treatments based on chemotherapy or radiotherapy are responsible for the occurrence of a gastrointestinal inflammatory condition known as mucositis^1–3^. Chemotherapy drugs, including 5-fluorouracil (5-FU), irinotecan and methotrexate, present high toxicity to cells with high proliferation rates, such as intestinal epithelial cells lining the gut mucosa. These drugs lead to apoptosis of those cells generating significant damage to epithelial barrier integrity, which allows bacterial colonization, invasion and consecutive triggering of inflammatory processes^4,5^. The scientific community believed that the gut microbiota would play a secondary role in the pathogenesis of the disease limited to aggravating conditions, such as bacterial translocation. However, recent studies have been investigating the role of commensal intestinal microbes under an ecological perspective where diverse organisms occupy niches that are essential for the development of mucositis^6,7^. The gut microbiota that colonizes the epithelial barrier of the intestine is composed mostly of bacteria which contribute to many functions of the host, while some are referred to pathobionts being capable of acquiring pathogenic characteristics under intestinal ecology disturbance^6^. Interestingly, it has been shown that germ-free mice are more resistant to 5-FU-induced mucositis, which reinforces the hypothesis that the microbiota is essential for the disease development^5,8^. Several pre-clinical and clinical studies have reported modifications in fecal microbiota diversity and composition following chemotherapy^9–12^.

To date, the treatment of gastrointestinal mucositis relies mostly on antibiotics and analgesics administration and since the efficiency of current therapies in alleviating this pathology has been questioned, several research groups are currently investigating alternative rationales^13^. In this context, promising achievements with probiotics, mainly members of the Lactic Acid Bacteria (LAB) group, have been reported in animal models of mucositis and seems to be useful to maintain intestinal barrier function^14–21^. The use of recombinant LAB strains, such as the model *Lactococcus lactis*, for delivering biologically active molecules with anti-inflammatory properties have also been explored as an alternative therapy for the treatment of mucositis^13,22,23^. Currently, a study designed recombinant strains of *L*. *lactis* to produce Trefoil factor 1 (TFF-1), involved in the maintenance of epithelial barrier integrity, revealing promising outcomes in the treatment of oral mucositis patients in clinical trials^22,23^. In a previous study, our research group evaluated the beneficial effect of a recombinant strain of *L*. *lactis* NZ9000 producing an antimicrobial peptide (AMP) isolated from human, the Pancreatitis-associated Protein (PAP), into mice exposed to 5-FU^24^. This AMP is naturally secreted by Paneth cells in mammalian small intestines and seems to be involved in the protection of the host by killing harmful bacteria and preventing the microbe-driven inflammatory process^25^. This strategy was shown to be useful to prevent mucositis, although the role of PAP in the microbiota was never explored in this model. Considering that the protein could be associated with host epithelial cell surface protection against pro-inflammatory bacteria in the mucosa, we sought to investigate PAP effects in the intestinal microbiome of 5-FU-treated mice in a mucositis experimental model. As *L*. *lactis* NZ9000 has shown protective effects in the same model, we also addressed its effects on the gut microbiome as well.

## Results

**Figure 1:**
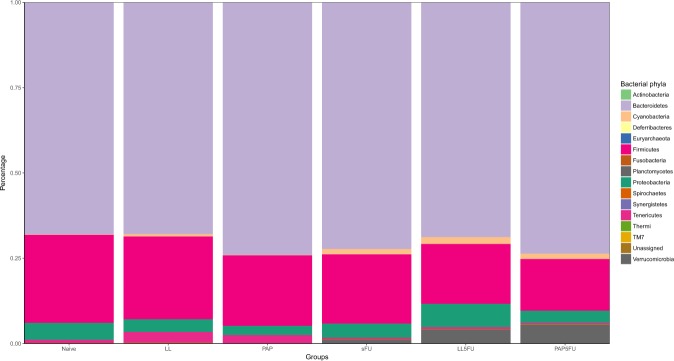
Phylum relative abundance in stool among the groups.

The high-throughput sequencing generated more than 135 megabases (Mb) for the enriched 16 rRNA gene V4 regions from all 72 samples, representing a total of 4.784.028 reads. Over 2.775.137 of the total reads from each sample passed quality control (Supplementary Table 1). The rarefaction curves approximated to a stable asymptote for all groups, meaning that the number of reads obtained was sufficient to represent the whole diversity in each group (Supplementary Figure 1). A total of fifteen phyla was obtained (Fig. 1) in all groups, and the most abundant were *Bacteroidetes* (70.8% ± 2.9%), *Firmicutes* (20.6% ± 4%), and *Proteobacteria* (4.3% ± 1.4%). From the 161 bacterial OTUs that were mapped to the database 1 belonged to *Synergistetes* phylum, 1 to *Thermus*, 1 to *Planctomycetes*, 1 to *Fusobacteria*, 1 to *Spirochaetes*, 2 to *Verrucomicrobia*, 2 to *Deferribacteres*, 2 to *Cyanobacteria*, 4 to *Tenericutes*, 8 to *Actinobacteria*, 28 to *Bacteriodetes*, 41 to *Proteobacteria* and 68 to *Firmicutes*. From 17 genus-level OTUs that were expected to be found in the Mock communities DNA samples, 15 and 14 were detected in the Mock even and Mock staggered respectively.

### 5-FU causes disruption in the microbial community structure when compared to the noninflamed groups

**Figure 2:**
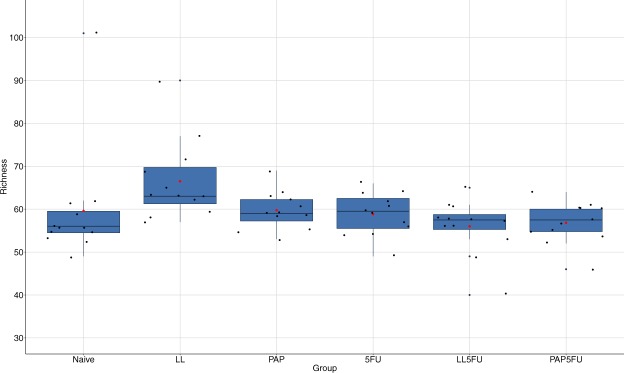
Microbial richness of the fecal microbiota among the groups. LL group showed an increase in richness when compared to Naïve (P = 0.014), LL5FU (P = 0.022) and PAP5FU (P = 0.028). Bonferroni, P-value <0,05.

**Figure 3:**
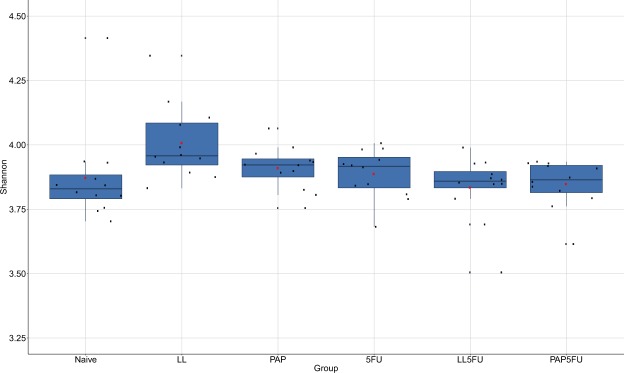
Diversity analysis of fecal microbiota among the groups. There was no significant statistical variation. Bonferroni, P-value <0,05.

**Figure 4:**
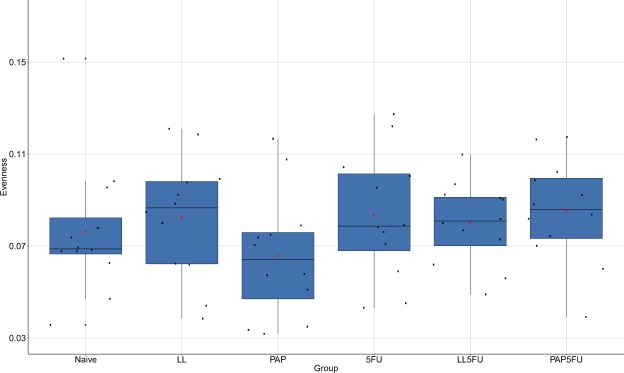
Microbial evenness of the fecal microbiota among the groups. There was no significant statistical variation. Bonferroni, P-value <0,05.

There were no statistically signifcant variation on richness (Fig. 2), Shannon (Fig. 3), and evenness (Fig. 4) among the groups, except LL group, which showed the highest richness when compared to Naïve (*P* = 0.014), LL5FU (*P* = 0.022) and PAP5FU (*P* = 0.028). However, the community structure of the naïve group was statistically different from the inflamed groups (5FU, LL5FU and PAP5FU). This dissimilarity was also observed when compared the LL and PAP to the inflamed groups (Table 1 and Table 2). Moreover, the group with inflamed animals that did not feed on probiotics (5FU group) showed highest values of the dissimilarity when compared to naïve, LL and PAP groups (R values in ANOSIM).

**Table 1:** Significant differences in microbial community among the groups using the multiple response permutation process (MRPP). The superior part of the table corresponds to the A-value and the inferior part to the P-value.

	Naive	LL	PAP	5FU	LL5FU	PAP5FU
Naive		0.029	−0.007	0.087	0.075	0.077
LL	0.103		0.012	0.122	0.038	0.011
PAP	0.517	0.229		0.131	0.068	0.063
5FU	**0**.**008**	**0**.**001**	**0**.**003**		0.025	0.054
LL5FU	**0**.**005**	**0**.**050**	**0**.**018**	0.159		−0.031
PAP5FU	**0**.**016**	0.245	**0**.**032**	**0**.**057**	0.986	

The A-value describes within-group homogeneity, compared to the random expectation. The P-values indicated the significant differences at the levels of P < 0.05.

**Table 2:** Significant differences in microbial community among the groups using ANOSIM. The superior part of the table corresponds to the R-value and the inferior part to the P-value.

	Naive	LL	PAP	5FU	LL5FU	PAP5FU
Naive		0.069	− 0.015	0.193	0.184	0.182
LL	0.073		0.006	0.272	0.099	0.029
PAP	0.562	0.331		0.244	0.141	0.106
5FU	**0**.**007**	**0**.**003**	**0**.**001**		0.074	0.109
LL5FU	**0**.**001**	**0**.**040**	**0**.**011**	0.095		−0.057
PAP5FU	**0**.**008**	0.185	**0**.**034**	0.063	0.954	

The P-values indicated the significant differences at the levels of P < 0.05.

### *L. lactis* NZ9000 and PAP are able to change the gut microbiota composition

There were no significant statistical differences in the phylum abundances between Naïve and LL group (Supplementary Figure 2); however, there was a significant decrease in the percentage of *Actinobacteria* (*P* = 0.003) in the animals fed with *L*. *lactis* expressing PAP when compared to the Naïve group.

**Figure 5:**
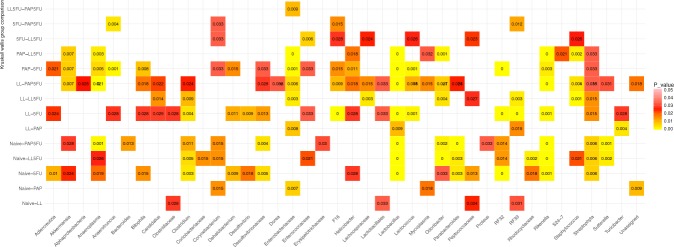
Significant changes of OTU proportion among the groups. Kruskall Wallis comparison. P-value <0,05.

When analyzed at the OTU level, LL group had a lower abundance of the OTU identified as *Clostridiaceae* and higher abundance of *Lactobacillales*, *Peptococcaceae*, and *RF39* than Naïve group (Fig. 5 and Supplementary Figure 3). The animals fed with the *L*. *lactis* expressing PAP showed a significant increase of *Mycoplasma* when compared to the naïve group. Moreover, mice receiving the recombinant strain showed decreased levels of *Enterobacteriaceae* and *Corynebacterium* compared to Naïve and *RF39*, *Turicibacter*, *Lactobacillus*, *and Enterobacteriaceae* in comparison to mice treated only with *LL* (Fig. 5 and Supplementary Figure 3).

### Mucositis induced by 5-Fluorouracil promotes a broad modification of the OTUs abundances in mice fecal microbiota

When the animals were inflamed, the abundance of *Actinobacteria* significantly decreased while the number of *Verrucomicrobia* increased when compared to Naïve (Supplementary Figure 2). Moreover, the OTUs *Adlercreutzia*, *Anaeroplasma*, *Clostridium*, *Helicobacter*, *Lactobacillus*, *Odoribacter*, *Rikenella* and *Streptophyta* significantly decreased when compared with the Naïve groups. At the same time, the following OTUs had the relative abundance increased in the inflamed animals: *Akkermansia*, *Bilophila*, *Dehalobacterium*, *Desulfovibrio*, *Desulfovibrionaceae*, *Parabacteroides*, *Peptococcaceae*, *RF32*, *Rhodocyclaceae* (Fig. 5 and Supplementary Figure 3).

### The administration of *L. lactis* NZ9000 maintain reduced levels of *Peptococcacea*, *Staphylococcus*, and *Corynebacterium* in inflamed mice

The animals that were fed with *L*. *lactis* and challenged with 5-FU (LL5FU) presented a significant increase in the relative abundances of the TM7 phylum (Supplementary Figure 2) and the OTUs identified as *F16*, *Enterobacteriaceae*, *Enterococaccea* and *Lactococcus* (Fig. 5 and Supplementary Figure 3). The *L*. *lactis* feeding treatment also decreased the proportion of *Corynebacterium*, *Lachnospiraceae*, *Peptococacceae*, and *Staphylococcus* which could be confirmed by the relative decreasing of the abundance of those OTUs in LL5FU when compared with 5FU group (Fig. 5 and Supplementary Figure 3).

### *L*. *lactis* expressing PAP restore the abundance of *RF39* and maintain reduced levels of *Anaerotruncus* and *Enterobacteriaceae* in mice with mucositis

The abundance of TM7 phylum was higher in PAP5FU than in 5FU group (Supplementary Figure 2). Abundance analysis at the OTU level shows that F16 and *RF39* increased while *Anaerotruncus* significantly decreased in the PAP5FU when compared to mice receiving only 5-FU (Fig. 5 and Supplementary Figure 3). Besides, treatment with PAP was responsible for reducing *Enterobacteriaceae* in comparison to mice fed only with LL (Fig. 5 and Supplementary Figure 3).

## Discussion

Recently, the interest in using probiotics for alleviating intestinal inflammation in patients submitted to antineoplastic chemotherapy has increased^15,26,27^. Although several works demonstrate the protective effects of probiotics in preventing mucositis, their impact on the microbial community structure has been poorly explored. In this study, we evaluated the effects of two *L*. *lactis* strains with anti-inflammatory properties, NZ9000 and LL-PAP^24,28^, on the intestinal microbial community structure through a 16 S rRNA gene metataxonomic analysis in a mouse model of mucositis. Three major phyla constituted the predominant gut microbiota in all mice from all experimental groups, consistent with previous surveys done with mammalian subjects^29^. In addition, almost all the genera constituting the Mock communities were detected. Although this finding was expected, it gives us more reliability when comparing our data with other related studies.

The 5-FU administration can cause a disturbance in the community structure, usually decreasing the richness and abundance of OTUs^30^. We were expecting to observe a similar effect in the inflamed animals, and despite the richness and diversity indices did not vary among the groups after 5-FU injection, we observed a significant alteration in the microbial community structure when comparing the noninflamed to the inflamed groups as indicated by the dissimilarity analyses. These findings suggest that different 5-FU regimens might cause disturbance states with particular structural traits.

At phylum level analysis, the proportion of *Verrucomicrobia* presented significant changes after 5-FU injection, corroborating with previous studies^31,32^. There are very few species belonging to this phylum found in the gut to date. The most dominant is *Akkermansia muciniphila*, a bacterium that scavenges mucins as a carbon and nitrogen source which has been inversely associated with obesity and diabetes, and presents protective activity in DSS-colitis in mice^33^. In the work of Kang and colleagues, extracted vesicles from *A*. *muciniphila* ameliorated inflammatory damage in the colon and reduced the expression of pro-inflammatory IL-6 stimulated by *E*. *coli*. Interestingly, we identified a genus-level OTU belonging to *Verrucomicrobia*, assigned as *Akkermansia*, being solely responsible for the increased proportion of *Verrucomicrobia* in the inflamed groups. The treatment with *L*. *lactis* presented an enrichment of *Akkermansia* compared to mice receiving 5-FU, although it was not statistically significant.

*Actinobacteria* was less enriched in the groups submitted to 5-FU injection compared to naive control. Intriguingly, this phylum was also decreased in healthy mice after treatment with *L*. *lactis* secreting PAP. Since *Actinobacteria* is almost exclusively formed by Gram-positive bacteria, its reduced abundance could be due to PAP anti-microbial affinity against Gram-positive bacteria as previously described^34,35^. However, there are controversies in the literature regarding the proportion of *Actinobacteria* in response to inflammation making it challenging to unravel its biological implications. For example, *Bifidobacterium spp* are considered as dominant bugs presenting anti-inflammatory properties, but other genera probably might play essential roles in the gut as well^10,36^. In our work, we identified two genera of *Actinobacteria* being significantly influenced during mucositis. A reduced proportion of *Adlercreutzia* was observed in mice receiving 5-FU suggesting its niche is essential for avoiding a dysbiosis state. The decreased abundance of *Adlercreutzia* has been previously reported in cases of colitis patients compared to control group, and in another clinical study, patients have shown a reduced proportion after chemotherapy submission^10^. Interestingly, this genus is currently formed by one species, *A*. *equolifaciens*, which produces an isoflavone metabolite, named equol, with anti-cancerous and anti-inflammatory properties^37,38^. Moreover, studies reveal that equol is exclusively produced by the intestinal microbiota^39^. The treatment with PAP did not cause any alteration at OTU level regarding *Actinobacteria*. The consumption of *L*. *lactis* culture did not seem to significantly increase the abundance of *Adlercreutzia* as well, but caused a reduction of another genus identified as *Corynebacterium*. In humans, these Gram-positive bacteria are commonly found on the skin, and some members of the genus are opportunistic pathogens when colonizing other sites of the body, such as the oral cavity. Commensal species of *corynebacteria* found in the gut are acquired from the mother’s skin mainly in cesarian*-*section infants^29^. Opportunistic *Corynebacterium* spp. have also been isolated from oral mucositis patients^40,41^. It is possible that competitive exclusion activity from *L*. *lactis* in reducing the abundance of *corynebacteria* might affect the dysbiosis state. However, their role in the gut requires further investigation.

In a similar context, there is no previous report about *Tenericutes* traits as indicators of health state in mucositis. Unclassified *RF39* and *Anaeroplasma* were found decreased in inflamed mice, treated only with 5-FU, while delivery of PAP was able to restore RF39 to normal levels.

The candidate phylum TM7 is a recently described subgroup of Gram-positive uncultivable bacteria initially found in different natural environmental habitats^42,43^. In our study, we identified a TM7 family-level OTU assigned as F16. Mice receiving 5-FU did not show alteration while treatment with *L*. *lactis* has caused significant enrichment of TM7/F16 at phylum- and OTU-level respectively. Controversially, Li and colleagues found a reduced abundance of TM7 proportion in mice treated with 5-FU in mice^32^. Although no definite correlation of TM7 has been associated with 5-FU-induced mucositis up to date, previous studies show TM7 OTUs has been associated with the pathogenesis of periodontitis^44^. Similarly, another study investigated TM7 in inflammatory bowel diseases (IBDs) suggesting it might play a key role in the development of inflammation^45^. Therefore, targeting TM7/F16 is of extreme importance to be investigated in further studies to improve or develop novel strategies for treating the disease.

No alteration was detected for *Firmicutes*, *Bacteriodetes*, and *Proteobacteria* at phylum-level analysis, possibly because they present the highest richness of OTUs being either down- or up-modulated. Perhaps they are more ecologically stable when compared to subdominant phyla such as *Verrucomicrobia*, TM7, and *Actinobacteria*, which presented significant changes after treatment with 5-FU or the *Lactococci* strains.

*Bacteriodetes* phylum comprised two members, *Rikenella* and *Odoribacter*, that were found decreased after 5-FU injection. *Odoribacter spp* are considered atypical opportunistic commensals because they produce butyrate and their presences are essential for preventing diseases such as hypertension though they may also contribute to intestinal abscesses^46^. Low levels of the *Odoribacter* population has been found in IBD patients^47^. However, the association of *Rikenella* with bad or good prognosis for inflammatory diseases has not been reported yet. Another *Bacteriodetes*, assigned as *Parabacteroides*, was found enriched in inflamed mice. *Parabacteroides* spp. are essential for digesting high-fiber diets that humans cannot process, and they tend to be missing from the gut of patients suffering from IBD^48^. *L*. *lactis* or PAP treatment in mice did not significantly affect any *Bacteriodetes* OTU either in healthy or inflamed mice, suggesting this phylum may present robustness against PAP inhibitory property and is less susceptible to *L*. *lactis* effects in the gut.

*Firmicutes* phylum is mainly formed by Gram-positive species of bacteria occupying several niches in the intestines, such as the production of Short-chain fatty acids (SCFAs) and trophic functions, although some are considered pathobionts as well^49,50^. Within *Firmicutes*, we verified that the majority of modulatory effects occurred in the *Clostridialles*, *Lactobacillales* and *Bacillales*. The *Lactobacillales* order, which is virtually formed by many species of bacteria with anti-inflammatory properties^51–53^, was found enriched in healthy mice that fed on *L*. *lactis* NZ9000 culture but seemed to be partially abrogated by 5-FU activity. The decrease of *Lactobacillus* corroborates with the study of Florez and colleagues, in which authors suggest that LAB species are more susceptible to 5-FU effects than other intestinal bacteria^54^. Although the treatment with *L*. *lactis* NZ9000 did not restore the abundance of *Lactobacillus* during mucositis, it caused an increase of the genus *Lactococcus*. Despite *Lactococcus spp*. are not usually considered to be commensal, this OTU was found in all groups, including mice that did not feed on the cultures containing live *Lactococci* strains.

The *Lactobacillales* order also contains opportunistic bacteria including *Streptococcus spp* and *Enterococcus spp*. In our work, unclassified *Enterococaccea* were found increased in inflamed mice treated with *L*. *lactis* NZ9000. In a recent study, *Enterococcaceae* dominance was associated with higher risk of neutropenia and diarrheal illness after chemotherapy treatment^55^. We were expecting a decrease of *Enterococcaceae* in mice treated with PAP, as we have previously demonstrated it was able to inhibit a representative commensal from this family, *E*. *faecalis*, *in vitro*,^24^. For unknown reasons, in the present study, we observed reduced levels of *Enterococcaceae* in PAP-treated mice, but it was not statistically significant when compared to the treatment with *L*. *lactis* NZ9000.

The *Bacillales* member *Staphylococcus* was decreased in mice consuming *L*. *lactis* wild-type strain. In humans, this genus comprises opportunistic commensals colonizing the skin and mucosal surfaces lining the nose and ear cavities. Studies suggest that parental transmission is the most common form for infants-gut colonization^56^. A study revealed that different *Staphylococcus* spp. strains had been isolated from the mouth of chemotherapy patients, presenting the ability to produce several staphylococcal enterotoxins^57^. These bacteria have also been reported to contribute to systemic infections during oral mucositis^58^. The representative species *S*. *aureus* caused 30 of 438 cases of bacteremia in neutropenic patients with cancer during a 10-year study period and septic metastases were more frequent in patients with *S*. *aureus* bacteremia, remaining as a significant cause of morbidity and mortality^59^. Therefore, our results imply a vital role for *L*. *lactis* in the prevention of *Staphylococcus* infection.

Our results suggest *Clostridium* was found depleted in mice submitted to 5-FU administration. The genus *Clostridium* comprises more than 200 species of bacteria in which some of these are pathogenic, but the majority is inoffensive. The representative pathogen is *C*. *difficile*, a Gram-positive bacteria that have been reported to be involved in IBD pathogenesis, but also in patients receiving antineoplastic chemotherapy^60^. Other *Clostridiales* bugs such as *Dehalobacterium* and unclassified *Peptococcaceae* was found overrepresented in mice injected with 5-FU. Although their role in the gut microbiome are unknown, the group of inflamed animals that consumed *L*. *lactis* NZ9000 culture restored the level of *Peptococcacea* and caused a reduction of the *Lachnospiraceae* population. Further studies are needed to provide possible clues about their biological implications. Another *Clostridiales* OTU which was suppressed by *L*. *lactis* consumption was *Anaerotruncus*, a recently described rod-like anaerobic bacterial genus belonging to *Clostridiaceae* family. The representative species is *A*. *colihominis* which have been isolated from human feces and associated with nosocomial bacteremia and to inflammatory traits in elderly subjects^61–63^. The expansion of *Proteobacteria* in the intestinal lumen, mainly *Enterobacteriaceae* has been consensually considered as a microbial signature of dysbiosis^64–66^. Among the *Proteobacteria* having increased numbers of mice receiving 5-FU, we identified three potential sulfate-reducing bacteria (SRB), *Desulfovibrio* spp., *Bilophila* spp. and unclassified *Desulfovibronaceae*. Increased levels of *Desulfovibrionacea* have also been found in ulcerative colitis [72,73] while *Bilophila wadsworthia*^67^ have been isolated in clinical intestinal infections and bacteremia. Several studies suggest that SRB acquire sulfate by depolymerization and desulphation of host mucus glycoproteins such as mucins, which are secreted by goblet cells lining the gastrointestinal tract^68^. In this context, SRB might act in intestinal disorders by secreting metabolic end products such as hydrogen sulfide, which inhibits the production of SCFAs by other commensal bacteria and by promoting the formation of Reactive Oxygen Species (ROS) and Reactive Nitrogen Species (RNS) (Loubinoux *et al*., 2002). While ROS/RNS may exacerbate the inflammatory process, they also serve as metabolic substrates for providing ATP for opportunistic *Enterobacteriaceae* in the gut^69^. The increase of *Enterobacteriaceae* family in the intestinal microbiota is associated with several intestinal disorders such as the Inflammatory Bowel Diseases and Colorectal Cancer. Moreover, recent studies reveal that the presence of *Escherichia coli*, the representative commensal species belonging to this family, can aggravate mucositis in mice^70^. Unexpectedly, in our study, feeding mice with *L*. *lactis* NZ9000 seems to favor the growth of this OTU in the inflamed mice. This result reiterate the importance of investigating the effects of probiotic strains in the gut microbiome as they may also stimulate the growth of undesirable bacteria. Previous studies have demonstrated that lactate, produced by LAB species can be used as an electron donor and may serve as a substrate for *Enterobacteria*^71^. In this context, as we did not observe significative augmentation of this OTU in healthy mice that also fed on *L*. *lactis* NZ9000 culture, we suggest lactate and the ROS/RNS generated by 5-FU activity in the mucosa might be acting synergically to provide fitness for *Enterobacteriaceae*^72^. In our previous work, we show that *L*. *lactis* NZ9000 producing PAP was able to preserve villous architecture of mice and increase Paneth cells activity in response to 5-FU inflammation^24^. Interestingly, in the present study our results show PAP delivery drastically inhibited the growth of the *Enterobacteriaceae* both in healthy and inflamed mice, suggesting a crucial protective role in the intestinal mucosa against the colonization of potential opportunistic Enterobacteria. Moreover, our study reinforces that PAP antimicrobial activity is not exclusively against Gram-positive bacteria. In an attempt to aggregate biological meaning to OTUs in which their role in the gut is not well established, we tried correlating the relative abundance with the metadata regarding inflammation markers that were assessed in our late work^24^. However, no significant correlation was obtained (data not shown).

## Conclusions

This study was the first step in characterizing the effects of the *L*. *lactis* NZ9000 and PAP-secreting strain in the prevention of 5-FU-induced dysbiosis. We demonstrate that both *Lactococci* strains were able to prevent specific niches being occupied by microorganisms with potential implications in the prognostic of mucositis. We believe the data generated in the study will be of extreme importance for improving therapeutic strategies for treating the disease.

## Methods

### Bacterial strains and growth conditions

*Lactococcus lactis* NZ9000 strain harboring pSEC:PAP vector (LL-PAP) and *L*. *lactis* NZ9000 strain carrying pSEC vector without the open reading frame of PAP (LL), were grown in M17 medium (Difco) supplemented with 0.5% glucose (GM17) at 30 °C without shaking. The strains were selected by the addition of chloramphenicol (Cm, 10 μg/mL). For nisin-induced PAP expression, LL-PAP was cultivated until the optical density at 600 nm reached 0.6. Afterward, 10 ng/mL of nisin (Sigma) were added to the medium and cultures were maintained at 30 °C for 2 h. Immediately after incubation, bacterial cells were washed with saline solution by centrifugation at 12000 rpm for 10 minutes to eliminate residual antibiotic compounds. *L*. *lactis* NZ9000 or LL-PAP cells were then dissolved in M17 without the addition of antibiotics and transferred to feeding bottles before experimentation.

### Animals and experimental treatment of the groups

Conventional female BALB/c mice between 6 and 8 weeks of age were obtained at Federal University of Minas Gerais (UFMG–Belo Horizonte, Brazil) and the Brazilian Ethics Committee on Animal Use (CEUA) approved the study. All mice were housed in cages in a controlled environment (23 °C, 12-/12-light/dark cycle with lighting), fed with standard chow diet, and provided with filtered water *ad libitum* before the experiment.

The animals were divided into six experimental groups (*n* = 4 in each group/cage), fed with standard chow diet and were administrated with 5 mL of filtered water or M17 medium containing 2.5 × 10^9^ CFU/mL of the following of bacterial strains: *L*. *lactis* NZ9000 or *L*. *lactis* expressing PAP by continuous feeding for 13 days^73^. For the induction of mucositis, 300 mg/Kg of 5-Fluorouracil (Flaudfluor) was administered intraperitoneally to mice on day 10. All mice were euthanized on day 14, and stools samples were collected and kept at −80°C.

The first three groups consisted of noninflamed mice: (i) Control, injected with 0.9% saline on day 10 and daily administered with water; (ii) LL, fed with *L*. *lactis* NZ9000; and (iii) LL-PAP, fed with *L*. *lactis* expressing PAP. The following groups were composed by those mice with mucositis: (iv) 5-FU, receiving filtered water; (v) LL-5FU, fed with *L*. *lactis* NZ9000, and (vi) LL-PAP, fed with *L*. *lactis* expressing PAP. All experiments were done in three replicates, totalizing 12 animals per group.

### 16S rRNA gene sequencing

Total DNA was extracted from 100 mg stool samples following QIAamp DNA Stool Mini Kit protocol (Cat No./ID: 51504, Qiagen) and quantified with Qubit®2.0 Fluorometer and Qubit® dsDNA BR Assay Kit (Life Technologies). The hypervariable V4 region from 16 S rRNA gene was amplified using fusion primers F515 (5′-GTGCCAGCMGCCGCGGTAA-3′) and R806 (5′-GGACTACHVGGGTWTCTAAT-3′)^74,75^. Sample emulsion PCR, emulsion breaking, and enrichment were performed using the Ion PGM™ Hi-Q™ View OT2 Kit (#A29900) according to the manufacturer’s instructions (Supplementary Document 1).

To determine the quality of metataxonomic method, two synthetic 16 S rRNA gene microbial communities (Mock Communities) of species with known genomes were used (Supplementary Document 1).

### Bioinformatics analyses for taxonomic assignment

Fastq file with raw data of all barcodes (expects two barcodes with mock communities) were used in OTU classification pipeline derived from 16 S rRNA gene profiling data analysis of Brazilian Microbiome Project^76^. Briefly, the raw fastq file was processed to strip barcodes using Usearch package^77^. Then, quality filtering was performed including removal of truncated and low-quality sequences (Phred score smaller than 20). Next, sequences were submitted to dereplication, abundance sorting, singleton removal, OTU clustering (97% similarity), and chimera filtering using Vsearch^78^. Finally, pre-processed sequences were assigned taxonomically using QIIME requiring 97% of sequence similarity threshold against the Greengenes 13.8 database^79^. The two barcodes with mock communities were processed using the same steps.

### Ecological analysis

The alpha diversity was estimated by richness, Shannon diversity and evenness index. The diversity was estimated using Shannon (*H′*) index (*H′* = *−Σni/n ln* (*ni/n*), where *ni* is the number of individuals in the taxon *i* and *n* is the total number of individuals), which is a heterogeneity index, influenced by both species richness and evenness. The evenness of species diversity was calculated using the Pielou formula: *H′/H′*max, where *H′* = Shannon index and *H′*max = the possible maximum diversity of the number of species (S) present in the community, defined by the formula *H′*max = l*n* S. Rarefaction curves was performed to indicate if the sequencing depth was sufficient to wholly capture the diversity present using iNEXT package^80,81^.

Two different non-parametric analyses were used to determine the significance of differences among the groups: analysis of similarity (ANOSIM) and multiresponse permutation procedure (MRPP)^82^ using Jaccard distance. A Bonferroni correction was applied to a *p*-value of 0.05 resulting in a significance level set at *P* = 0.0033.

### Ethics approval

The Protocol no. 366/2012, related to the present project is in agreement with the Ethical Principles in Animal Experimentation, adopted by the Ethics Committee in Animal Experimentation (CEUA/UFMG), and was approved on 11/04/2013.

## Electronic supplementary material

Supplementary Documents

Supplementary Dataset 1

Supplementary Dataset 2

## Data Availability

The datasets generated and/or analyzed during the current study are available in the Figshare repository, https://figshare.com/s/057a1c06772fbf6de6a9. Metadata, the OTU table, pipelines and scripts have all been included as supplementary materials.
