# Supplementary material for: Gut microbiome modulation during treatment of mucositis with the dairy bacterium Lactococcus lactis and recombinant strain secreting human antimicrobial PAP
Source: Sci Rep. 2018 Oct 10;8:15072. doi: 10.1038/s41598-018-33469-w (PMC6180057; doi:10.1038/s41598-018-33469-w)
Supplement: Supplementary file 1 — Supplementary Documents [file 41598_2018_33469_MOESM1_ESM.pdf]

**Gut microbiome modulation during treatment of mucositis with the dairy bacterium *Lactococcus lactis* and recombinant strain secreting human antimicrobial PAP**

Rodrigo Carvalho[1*], Aline Vaz[1], Felipe Luiz Pereira[1], Fernanda Dorella[1], Eric Aguiar[2], Jean-Marc Chatel[4], Luis Bermudez[4], Philippe Langella[4], Gabriel Fernandes[3], Henrique Figueiredo[1], Aristóteles Goes-Neto[1], Vasco Azevedo[1]

[1]*Federal University of Minas Gerais (UFMG-ICB), Belo Horizonte, MG, Brazil*

[2]*Federal University of Bahia (UFBA), Salvador, BA, Brazil*

[3]*Fiocruz - Centro de Pesquisa Renê Rachou - Belo Horizonte, MG, Brazil*

[4]*Micalis Institute, INRA, AgroParisTech, Université Paris-Saclay - Jouy-en-Josas, France*

*Corresponding author:
Rodrigo Dias de Oliveira Carvalho
rodrigodoc2@gmail.com
Tel/FAX 00 55 31 3409 2610
UFMG, Av. Antônio Carlos, 6627, Belo Horizonte, MG, Brasil. CEP 31270-901

# Suplementary Documents

## Setting up the environment

```
http://www.brmicrobiome.org/16siontorrent

https://conda.io/miniconda.html

wget https://repo.continuum.io/miniconda/Miniconda2-latest-Linux-x86_64.sh

http://qiime.org/install/install.html

conda install numpy

conda create -n qiime1 python=2.7 qiime matplotlib=1.4.3 mock nose -c
bioconda

source activate qiime1

source deactivate
```

## Getting auxiliary scripts

```
https://github.com/vpylro/BMP
git clone https://github.com/vpylro/BMP.git
```

## Installing VSEARCH program and scripts

```
###### vsearch https://github.com/torognes/vsearch
wget https://github.com/torognes/vsearch/archive/v2.6.0.tar.gz
tar xzf v2.6.0.tar.gz
cd vsearch-2.6.0
./autogen.sh
./configure
make

###scripts for vsearch use [derived from usearch]
https://drive5.com/python/
wget https://drive5.com/python/python_scripts.tar.gz
tar -zxvf python_scripts.tar.gz

###auxiliar scripts
wget http://drive5.com/python/python_scripts.tar.gz
```

## Pre-processing

```
#####
>python2.7 ../scripts/fastq_strip_barcode_relabel2.py all.basecaller.fastq
GTGCCAGCNGCCGCGGTAA barcodesIon.fasta labmcf > reads.stripped.fastq
  5896231 seqs
   4931985 matched
    787439 barcode mismatches
    176807 primer mismatches

>../scripts/vsearch/bin/vsearch  --fastx_filter reads.stripped.fastq --
fastq_maxee 1.0 --fastq_trunclen 200 --fastaout
reads.stripped.filtered.fasta
vsearch v2.6.0_linux_x86_64, 220.2GB RAM, 70 cores
https://github.com/torognes/vsearch

Reading input file 100%
```

```
2890095 sequences kept (of which 2889774 truncated), 2041890 sequences
discarded.

>../scripts/vsearch/bin/vsearch --derep_fulllength
reads.stripped.filtered.fasta --output reads.stripped.filtered.derep.fasta
--sizeout
vsearch v2.6.0_linux_x86_64, 220.2GB RAM, 70 cores
https://github.com/torognes/vsearch

Reading file reads.stripped.filtered.fasta 100%
578019000 nt in 2890095 seqs, min 200, max 200, avg 200
Dereplicating 100%
Sorting 100%
330969 unique sequences, avg cluster 8.7, median 1, max 140804
Writing output file 100%

> ../scripts/vsearch/bin/vsearch --sortbysize
reads.stripped.filtered.derep.fasta --output
reads.stripped.filtered.derep.sorted.fasta --minsize 2
vsearch v2.6.0_linux_x86_64, 220.2GB RAM, 70 cores
https://github.com/torognes/vsearch

Reading file reads.stripped.filtered.derep.fasta 100%
66193800 nt in 330969 seqs, min 200, max 200, avg 200
Getting sizes 100%
Sorting 100%
Median abundance: 3
Writing output 100%



>../scripts/vsearch/bin/vsearch --cluster_size
reads.stripped.filtered.derep.sorted.fasta --consout
reads.stripped.filtered.derep.sorted.OTUs.fasta --id 0.97
vsearch v2.6.0_linux_x86_64, 220.2GB RAM, 70 cores
https://github.com/torognes/vsearch

Reading file reads.stripped.filtered.derep.sorted.fasta 100%
16280800 nt in 81404 seqs, min 200, max 200, avg 200
Masking 100%
Sorting by abundance 100%
Counting k-mers 100%
Clustering 100%
Sorting clusters 100%
Writing clusters 100%
Clusters: 3939 Size min 1, max 2783, avg 20.7
Singletons: 1452, 1.8% of seqs, 36.9% of clusters
Multiple alignments 100%


>../scripts/vsearch/bin/vsearch --usearch_global
reads.stripped.filtered.fasta --db
reads.stripped.filtered.derep.sorted.OTUs.fasta --strand plus --id 0.97 --
uc map.uc
vsearch v2.6.0_linux_x86_64, 220.2GB RAM, 70 cores
https://github.com/torognes/vsearch

Reading file reads.stripped.filtered.derep.sorted.OTUs.fasta 100%
787804 nt in 3939 seqs, min 198, max 202, avg 200
Masking 100%
Counting k-mers 100%
```

```
Creating k-mer index 100%
Searching 100%
Matching query sequences: 2865422 of 2890095 (99.15%)
```

**Assigning Taxonomy**

```
##
assign_taxonomy.py -i reads.stripped.filtered.derep.sorted.OTUs.fasta -o
output
```

**Converting UC to OUT table**

```
python2.7 ../vsearch_scripts/uc2otutab.py map.uc  > otu_table.txt
map.uc 100.0%
```

**Home made script to create table with OTUs and counts**

```
##### converting OTU ID to taxon [considering level 5 - gender]
 convertOTUtable2Taxon.pl -table ../otu_table.txt -tax
reads.stripped.filtered.derep.sorted.OTUs_tax_assignments.txt -level 5 >
OUT_table_formatted.tab
```

**Library preparation and Sequencing**

The forward fusion primers contain the A sequence with barcode tags, and reverse fusion primer contains the trP1 sequence, their 5 -ends adjacent to the target-specific portions of the primers [Ion Amplicon Library Preparation (Fusion Method), Publication Number4468326, RevisionC]. Each PCR reaction contained the following components: 1x Platinum® PCR SuperMix High Fidelity; 5 μM of each oligonucleotide primer; approximately 20-50 ng genomic DNA and sterilized, deionized water. Cycle parameters were as follows: 1 initial denaturation cycle at 94°C for 3 min, followed by 40 denaturation cycles at 94°C for 30 sec, annealing at 58°C for 30 sec and extension at 68°C for 1 min/kb. PCR products were confirmed using QiAxcel Advanced System (Qiagen) and purified with Agencourt® AMPure XP Reagent (Beckman Coulter). Each amplicon was quantitate using Qubit®2.0 Fluorometer and Qubit® dsDNA HS Assay Kit (Life Technologies) and pooling in equimolar amounts proportion to produce a composite sample with a final concentration of 26pM.

Sample emulsion PCR, emulsion breaking, and enrichment were performed using the Ion PGM™ Hi-Q™ View OT2 Kit (#A29900) according to the manufacturer's instructions. Briefly, an input concentration of one DNA template copy/Ion Sphere Particle (ISP) was added to the emulsion PCR master mix, and the emulsion was generated using an OT2 (Life Technologies). Next, the ISPs were recovered, and Dynabeads MyOne Streptavidin C1 beads (Life Technologies) were used to enrich for template-positive ISPs. The sample was prepared for sequencing using the Ion PGM™ Hi-Q™ View Sequencing Kit (#A30044). Each composite sample was loaded onto an Ion 318 chip and sequenced on the PGM (Personal Genome Machine) system for 850 flows.

**Mock communities**

The first community, HMD-782D contains genomic DNA from 20 bacterial strains containing equimolar (Even) ribosomal RNA operon counts (100,000 copies per organism per μL. The second one is HMD783D, which contains genomic DNA from 20 bacterial strains containing staggered ribosomal RNA operon counts (1,000 to 1,000,000 copies per organism per μL). Both reagents were obtained from BEI Resources, NIAID, NIH as part of the Human Microbiome Project: Genomic DNA from Microbial Mock Community B (Even, Low Concentration), v5.1L, for 16S rRNA gene sequencing, HM-782D; and Genomic DNA from Microbial Mock

Community B (Staggered, Low Concentration), v5.2L, for 16S rRNA gene sequencing, HM-783D.

# Suplementary Figures and Tables

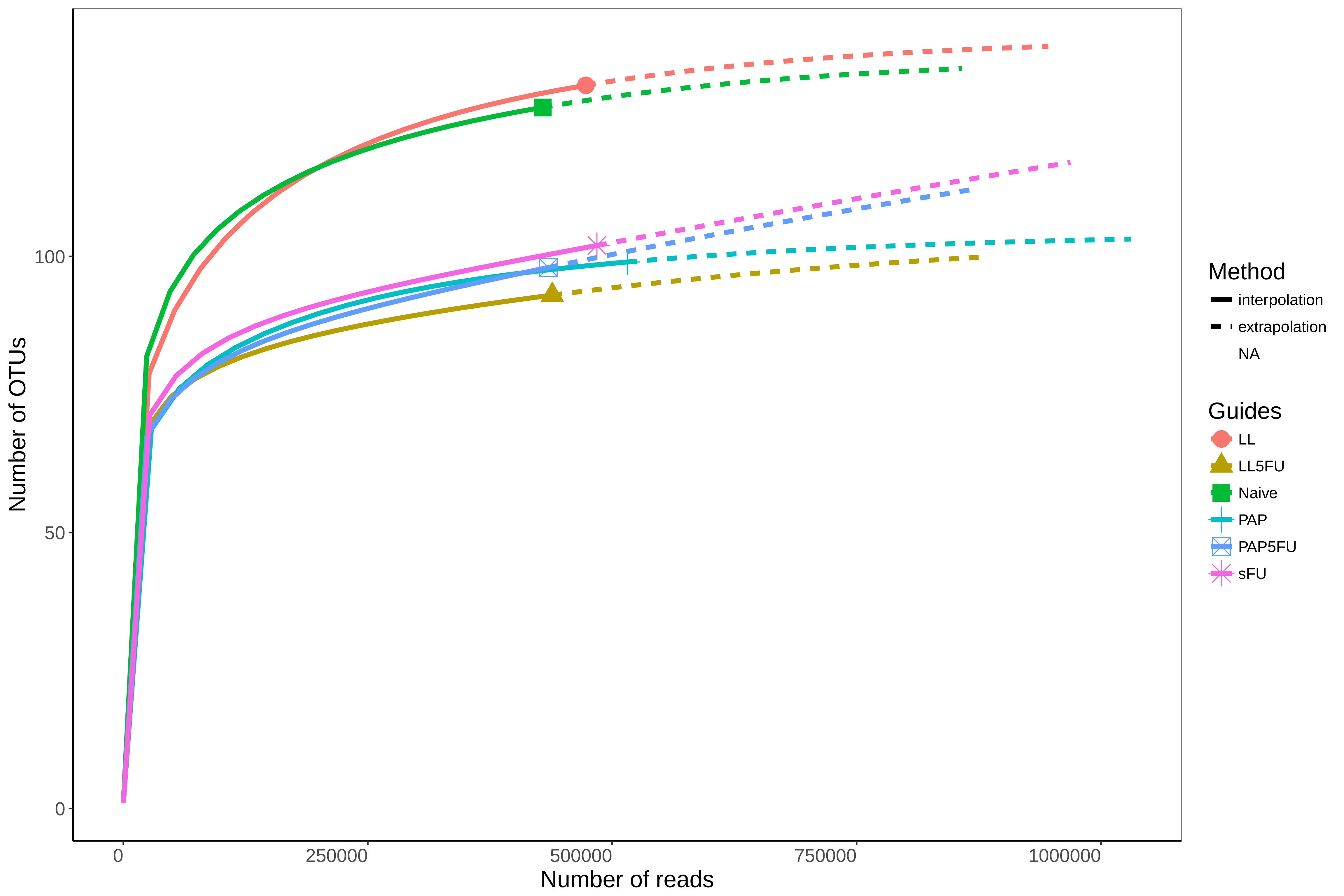
Number of OTUs
100
50
0
0
250000
500000
750000
1000000
Number of reads
Method
interpolation
extrapolation
NA
Guides
LL
LL5FU
Naive
PAP
PAP5FU
sFU

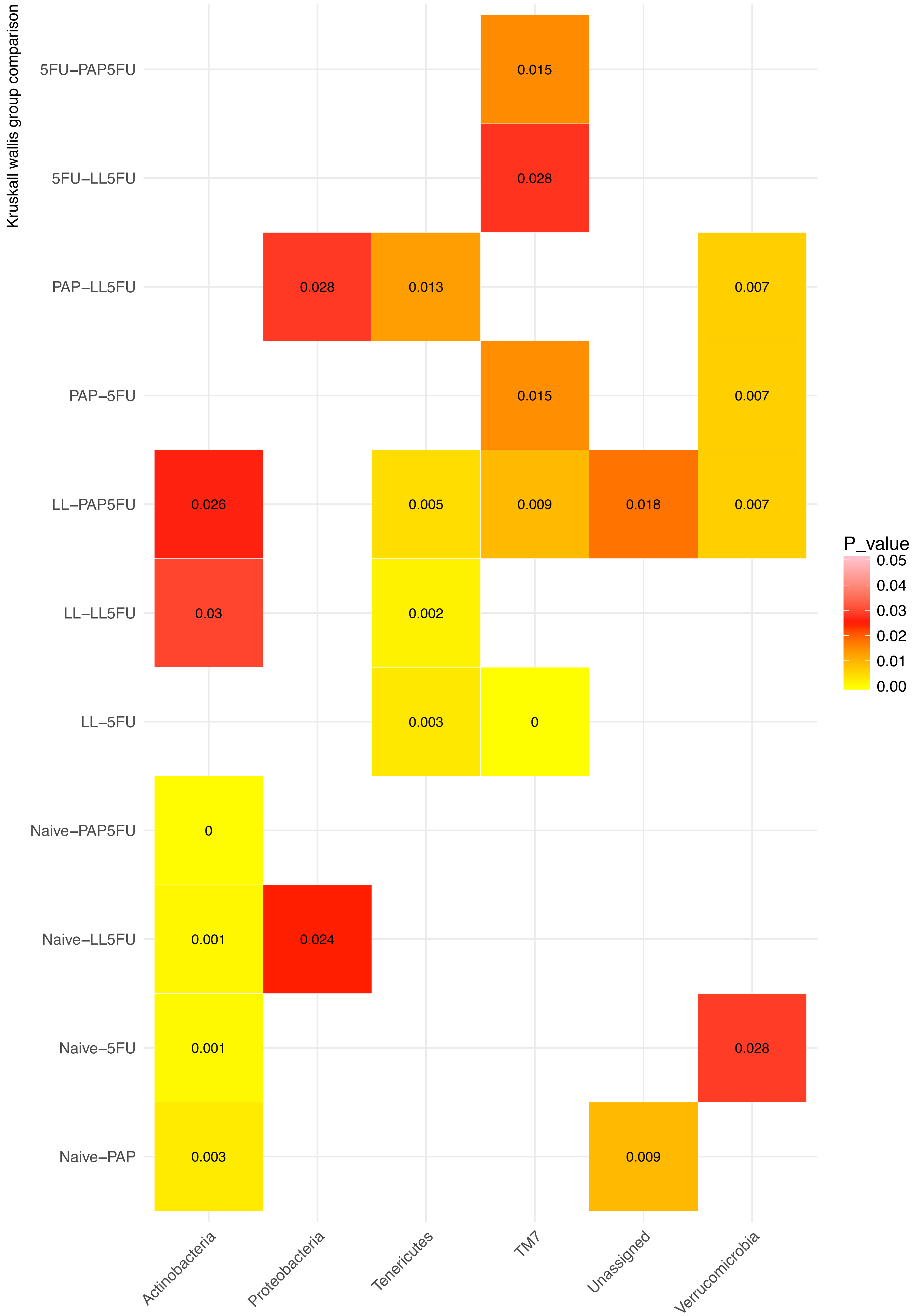

Kruskall wallis group comparison
5FU–PAP5FU
5FU–LL5FU
PAP–LL5FU
PAP–5FU
LL–PAP5FU
LL–LL5FU
LL–5FU
Naive–PAP5FU
Naive–LL5FU
Naive–5FU
Naive–PAP
Actinobacteria
Proteobacteria
Tenericutes
TM7
Unassigned
Verrucomicrobia
0.015
0.028
0.028
0.013
0.007
0.015
0.007
0.026
0.005
0.009
0.018
0.007
0.03
0.002
0.003
0
0
0.001
0.024
0.001
0.028
0.003
0.009
P_value
0.05
0.04
0.03
0.02
0.01
0.00

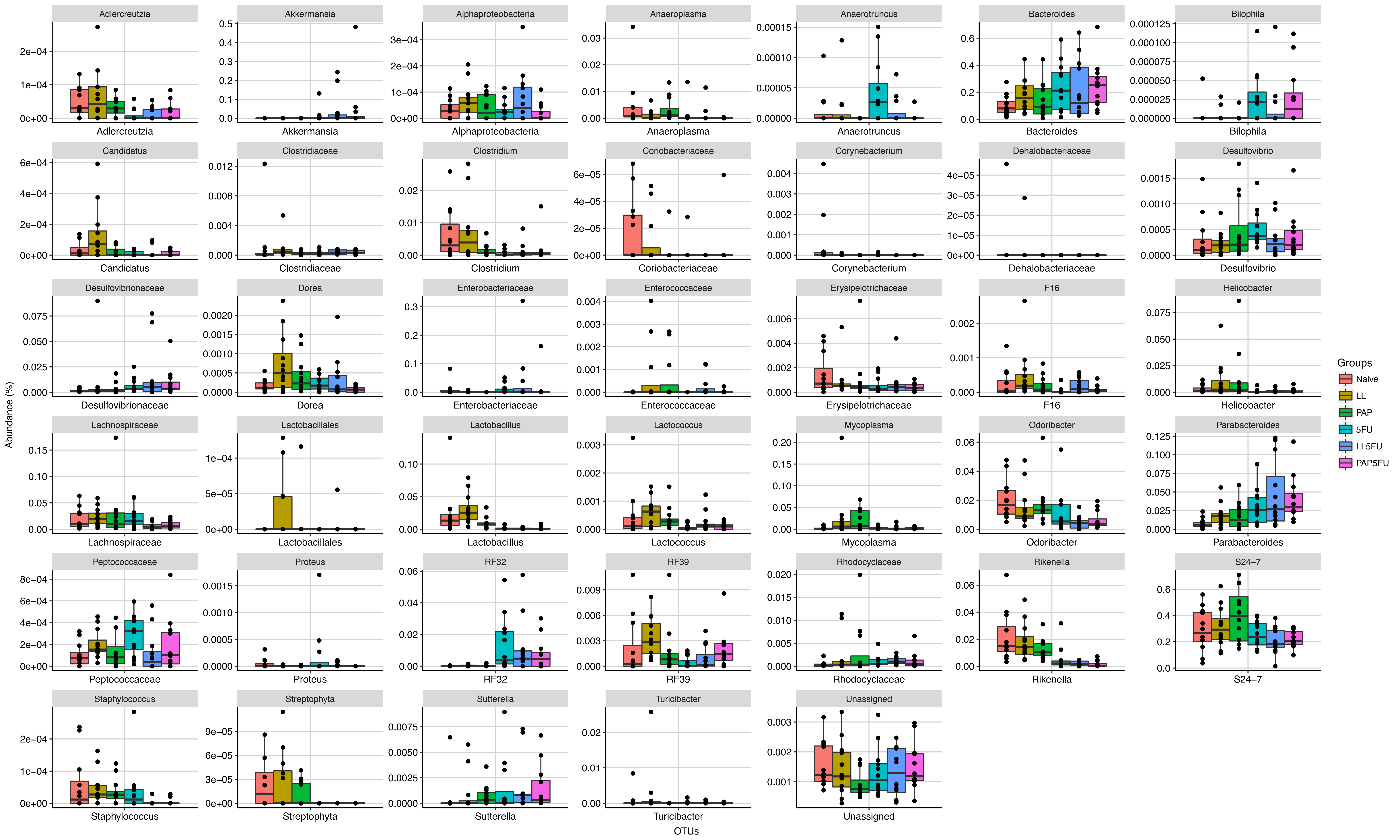
Adlercreutzia
Akkermansia
Alphaproteobacteria
Anaeroplasma
Anaerotruncus
Bacteroides
Bilophila
Candidatus
Clostridiaceae
Clostridium
Coriobacteriaceae
Corynebacterium
Dehalobacteriaceae
Desulfovibrio
Desulfovibrionaceae
Dorea
Enterobacteriaceae
Enterococcaceae
Erysipelotrichaceae
F16
Helicobacter
Lachnospiraceae
Lactobacillales
Lactobacillus
Lactococcus
Mycoplasma
Odoribacter
Parabacteroides
Peptococcaceae
Proteus
RF32
RF39
Rhodocyclaceae
Rikenella
S24−7
Staphylococcus
Streptophyta
Sutterella
Turicibacter
Unassigned
Abundance (%)
OTUs
Groups
Naive
LL
PAP
5FU
LL5FU
PAP5FU

**Supplemmentary Figure 1.** Rarefaction curves for all groups.

**Supplemmentary Figure 2.** Significant differences in Phylum proportion among the groups. Kruskall Wallis group comparison. P-value < 0,05.

**Supplemmentary Figure 3.** OTUs relative abundance in stool among the groups.

**Supplementary Table 1.** Reads filtering overview

| Samples | Total reads | Quality filtering, length truncate | Dereplication | Used for taxonomic assignment |
|---|---|---|---|---|
| Naive 1.1 | 57538 | 35233 | 3785 | 34960 |
| Naive 1.2 | 55101 | 29675 | 2863 | 26448 |
| Naive 1.3 | 52818 | 30578 | 2528 | 38115 |
| Naive 1.4 | 59293 | 35330 | 2662 | 29501 |
| Naive 2.1 | 51183 | 26732 | 3407 | 44401 |
| Naive 2.2 | 78267 | 44644 | 4305 | 43796 |
| Naive 2.3 | 50730 | 29523 | 3974 | 30464 |
| Naive 2.4 | 57798 | 35287 | 3167 | 29144 |
| Naive 3.1 | 68064 | 38859 | 6984 | 37973 |
| Naive 3.2 | 72247 | 44925 | 8277 | 35226 |
| Naive 3.3 | 64090 | 38156 | 3782 | 35126 |
| Naive 3.4 | 75795 | 43986 | 4394 | 43629 |
| LL 1.1 | 77548 | 46749 | 5112 | 46405 |
| LL 1.2 | 70737 | 43289 | 4375 | 35058 |
| LL 1.3 | 73041 | 39742 | 6935 | 21938 |
| LL 1.4 | 94146 | 56276 | 6527 | 42939 |
| LL 2.1 | 63240 | 35705 | 5469 | 36594 |
| LL 2.2 | 63716 | 36987 | 4697 | 35001 |
| LL 2.3 | 57172 | 34955 | 3740 | 38932 |
| LL 2.4 | 66255 | 40488 | 3985 | 34745 |
| LL 3.1 | 34871 | 22101 | 2529 | 31880 |
| LL 3.2 | 53214 | 35189 | 3372 | 55866 |
| LL 3.3 | 51965 | 32009 | 3011 | 40280 |
| LL 3.4 | 89737 | 54103 | 7705 | 53444 |
| PAP 1.1 | 69473 | 34532 | 6567 | 33732 |
| PAP 1.2 | 68990 | 34730 | 3171 | 61955 |
| PAP 1.3 | 54191 | 35527 | 3628 | 40997 |
| PAP 1.4 | 76530 | 48891 | 5453 | 34451 |
| PAP 2.1 | 106975 | 62713 | 8438 | 58906 |
| PAP 2.2 | 96199 | 59348 | 6811 | 38787 |
| PAP 2.3 | 63288 | 36476 | 5923 | 35276 |
| PAP 2.4 | 81394 | 51730 | 6102 | 35961 |
| PAP 3.1 | 69991 | 41261 | 4420 | 32794 |
| PAP 3.2 | 62435 | 38962 | 3831 | 48489 |
| PAP 3.3 | 52055 | 32975 | 3343 | 51381 |
| PAP 3.4 | 70788 | 42907 | 4044 | 42691 |
| 5FU 1.1 | 70387 | 40004 | 3940 | 39829 |
| 5FU 1.2 | 73089 | 41234 | 4685 | 35679 |
| 5FU 1.3 | 97206 | 52372 | 6551 | 54245 |
| 5FU 1.4 | 66334 | 37295 | 5367 | 40824 |
| 5FU 2.1 | 62309 | 35970 | 3956 | 35524 |
| 5FU 2.2 | 58120 | 35846 | 3648 | 35181 |
| 5FU 2.3 | 75568 | 43634 | 4551 | 51949 |
| 5FU 2.4 | 79498 | 49208 | 5544 | 43381 |
| 5FU 3.1 | 92244 | 54554 | 5301 | 22310 |
| 5FU 3.2 | 61823 | 35663 | 5071 | 36900 |
| 5FU 3.3 | 36045 | 22427 | 2341 | 48852 |
| 5FU 3.4 | 64627 | 39897 | 3255 | 39728 |
| LL5FU 1.1 | 26305 | 13894 | 1295 | 36029 |
| LL5FU 1.2 | 40226 | 27844 | 1540 | 13818 |
| LL5FU 1.3 | 72045 | 39627 | 3789 | 43019 |

| | | | | |
|---|---|---|---|---|
| LL5FU 1.4 | 65692 | 36239 | 3820 | 36768 |
| LL5FU 2.1 | 74597 | 43588 | 5695 | 27773 |
| LL5FU 2.2 | 58123 | 35401 | 5301 | 34803 |
| LL5FU 2.3 | 66442 | 41886 | 5555 | 37195 |
| LL5FU 2.4 | 52591 | 34489 | 3047 | 39434 |
| LL5FU 3.1 | 59193 | 37156 | 4893 | 41329 |
| LL5FU 3.2 | 64620 | 37394 | 2982 | 48392 |
| LL5FU 3.3 | 73079 | 48642 | 4944 | 34346 |
| LL5FU 3.4 | 84183 | 46150 | 4909 | 45844 |
| PAP5FU 1.1 | 67203 | 36326 | 3398 | 41765 |
| PAP5FU 1.2 | 69645 | 19991 | 2254 | 36144 |
| PAP5FU 1.3 | 28832 | 16951 | 2134 | 35352 |
| PAP5FU 1.4 | 73882 | 41962 | 4153 | 45831 |
| PAP5FU 2.1 | 60248 | 35487 | 3412 | 19815 |
| PAP5FU 2.2 | 68881 | 37041 | 4879 | 36591 |
| PAP5FU 2.3 | 62785 | 42851 | 2707 | 33299 |
| PAP5FU 2.4 | 63685 | 36314 | 5513 | 16813 |
| PAP5FU 3.1 | 82126 | 46175 | 4989 | 42692 |
| PAP5FU 3.2 | 64017 | 33664 | 4259 | 48315 |
| PAP5FU 3.3 | 78578 | 48800 | 5873 | 35752 |
| PAP5FU 3.4 | 78925 | 42682 | 4521 | 42331 |
| Mock 1 | 83972 | 49202 | 7421 | 48765 |
| Mock 2 | 63969 | 41660 | 4159 | 41518 |
